# The ‘Real-World Approach’ and Its Problems: A Critique of the Term Ecological Validity

**DOI:** 10.3389/fpsyg.2020.00721

**Published:** 2020-04-30

**Authors:** Gijs A. Holleman, Ignace T. C. Hooge, Chantal Kemner, Roy S. Hessels

**Affiliations:** ^1^Department of Experimental Psychology, Helmholtz Institute, Utrecht University, Utrecht, Netherlands; ^2^Department of Developmental Psychology, Utrecht University, Utrecht, Netherlands; ^3^Brain Center, University Medical Center Utrecht, Utrecht, Netherlands

**Keywords:** ecological validity, experiments, real-world approach, generalizability, definitions

## Abstract

A popular goal in psychological science is to understand human cognition and behavior in the ‘real-world.’ In contrast, researchers have typically conducted their research in experimental research settings, a.k.a. the ‘psychologist’s laboratory.’ Critics have often questioned whether psychology’s laboratory experiments permit generalizable results. This is known as the ‘real-world or the lab’-dilemma. To bridge the gap between lab and life, many researchers have called for experiments with more ‘ecological validity’ to ensure that experiments more closely resemble and generalize to the ‘real-world.’ However, researchers seldom explain what they mean with this term, nor how more ecological validity should be achieved. In our opinion, the popular concept of ecological validity is ill-formed, lacks specificity, and falls short of addressing the problem of generalizability. To move beyond the ‘real-world or the lab’-dilemma, we believe that researchers in psychological science should always specify the particular context of cognitive and behavioral functioning in which they are interested, instead of advocating that experiments should be more ‘ecologically valid’ in order to generalize to the ‘real-world.’ We believe this will be a more constructive way to uncover the context-specific and context-generic principles of cognition and behavior.

## Introduction

A popular goal in psychological science is to understand human cognition and behavior in the ‘real-world.’ In contrast, researchers have traditionally conducted experiments in specialized research settings, a.k.a. the ‘psychologist’s laboratory’ (Danziger, 1994; Hatfield, 2002). Over the course of psychology’s history, critics have often questioned whether psychology’s lab-based experiments permit the generalization of results beyond the laboratory settings within which these results are typically obtained. In response, many researchers have advocated for more ‘ecologically valid’ experiments, as opposed to the so-called ‘conventional’ laboratory methods (Neisser, 1976; Aanstoos, 1991; Kingstone et al., 2008; Shamay-Tsoory and Mendelsohn, 2019; Osborne-Crowley, 2020). In recent years, several technological advances (e.g., virtual reality, wearable eye trackers, mobile EEG devices, fNIRS, biosensors, etc.) have further galvanized researchers to emphasize the importance of studying human cognition and behavior in the ‘real-world,’ as new technologies will aid researchers in overcoming some of the inherent limitations of laboratory experiments (Schilbach, 2015; Shamay-Tsoory and Mendelsohn, 2019; Sonkusare et al., 2019).

In this article, we will argue that the general aspiration of researchers to understand human cognition and behavior in the ‘real-world’ by conducting experiments that are more ‘ecologically valid’ (henceforth referred to as the ‘real-world approach’) is not without its problems. Most notably, we will argue that the popular term ‘ecological validity,’ which is widely used nowadays by researchers to discuss whether experimental research resembles and generalizes to the ‘real-world,’ is shrouded in both conceptual and methodological confusion. As we ourselves are interested in cognitive and behavioral functioning in the context of people’s everyday experience, and conduct experiments across various ‘laboratory’ and ‘real-world’ environments, we have seen how the uncritical use of the term ‘ecological validity’ can lead to rather misleading and counterproductive discussions. This not only holds for how this concept is used in many scholarly articles and textbooks, but also in presentations and discussions of experimental research at conferences, during the review process, and when talking with students about experimental design and the analysis of evidence.

Although the usage of the term ecological validity has previously been criticized by several scholars (Hammond, 1998; Schmuckler, 2001; cf. Araujo et al., 2007; Dunlosky et al., 2009), we think that these critiques have largely been overlooked. Therefore, it will be necessary to cover some of the same ground. The contribution of this article is threefold. First, we extend the critique of the term ecological validity and apply it to the field of social attention. Second, we scrutinize some of the assumptions that guide the contemporary framework of ecological validity, specifically those regarding artificiality–naturality and simplicity–complexity. Finally, our article is meant to educate a new generation of students and researchers on the historical roots and conceptual issues of the term ecological validity. This article consists of four parts. First, we will provide a brief history of the so-called ‘real-world or the lab’-dilemma and discuss several definitions and interpretations of the term ecological validity. Second, we will go into the historical roots of the concept of ecological validity and describe how the original meaning of this concept has transformed significantly. Third, we will scrutinize the prevailing assumptions that seems to guide how researchers are currently using the term ecological validity. Finally, we will apply our conceptual analysis to a specific field of study, namely the field of social attention. In recent years, this field has been particularly concerned with issues of ecological validity and generalizability. Therefore, the field of social attention offers an exemplary case to explain how the uncritical use of the terms ‘ecological validity’ and the ‘real-world’ may lead to misleading and counterproductive conclusions.

## A Brief History of the ‘Real-World or the Lab’-Dilemma

The popular story of psychology (or the broader ‘cognitive sciences’) has it that *“psychology became a science by rising from the ‘armchair’ of speculation and uncontrolled observation, and entering the laboratory to undertake controlled observation and measurement”* (Hatfield, 2002, p. 208). The ‘psychologist’s laboratory’, a special room furnished with all kinds of lab paraphernalia and sophisticated equipment, has been regarded as the celebrated vehicle of psychology’s journey into sciencehood (Danziger, 1994; Goodwin, 2015). However, despite psychologists’ long tradition of laboratory experimentation (for a history and discussion, see Gillis and Schneider, 1966), there also have been many critical voices saying that psychology’s laboratory experiments are too limited in scope to study how people function in daily life. For example, Brunswik (1943, p. 262) once wrote that experimental psychology was limited to *“narrow-spanning problems of artificially isolated proximal or peripheral technicalities of mediation which are not representative of the larger patterns of life”.* Barker (1968, p. 3) wrote that *“it is impossible to create in the laboratory the frequency, duration, scope and magnitude of some important human conditions.”* Neisser (1976, p. 34) wrote that *“contemporary studies of cognitive processes usually use stimulus material that is abstract, discontinuous, and only marginally real.”* Bronfenbrenner (1977, p. 513) wrote that *“many of these experiments involve situations that are unfamiliar, artificial, and short-lived and that call for unusual behaviors that are difficult to generalize to other settings.”* Kingstone et al. (2008, p. 355) declared that *“the research performed in labs, and the findings they generate, are in principle and in practice unlikely to be of relevance to the more complex situations that people experience in everyday lif*e,*”* and Shamay-Tsoory and Mendelsohn (2019, p. 1) stated that “*conventional experimental psychological approaches have mainly focused on investigating behavior of individuals as isolated agents situated in artificial, sensory, and socially deprived environments, limiting our understanding of naturalistic cognitive, emotional, and social phenomena.”*

**FIGURE 1:**
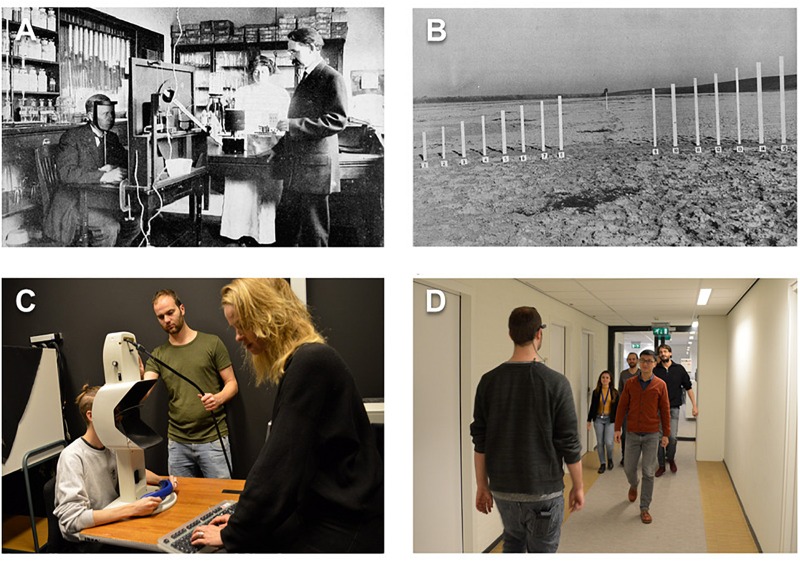
Examples of historical and contemporary laboratory rooms and field experiments. **(A)** A laboratory room from the early 20th century. A participant is seated in front a ‘disc tachistoscope,’ an apparatus to display visual images (adapted from Hilton, 1920). **(B)** A picture of a field experiment by J. J. Gibson. Observers had to judge the size of an object in the distance (adapted from Gibson, 1950). **(C)** A 21st century eye tracking laboratory. A participant is seated in front of a *SMI Hi-Speed* tower-mounted eye tracker (based on Valtakari et al., 2019). **(D)** A wearable eye-tracker (barely visible) is used to measure gaze behavior while participants walked through corridors with human crowds (Hessels et al., 2020). Copyright statement – Panels **(A,B)**. All photographs are used under the provision of the “fair use” U.S. Copyright Act 107 and Dutch Copyright Law Article 15a for non-profit purposes of research, education and scholarly comment. The photograph from W. Hilton’s book: *Applied Psychology: Driving Power of Thought* (Original date of publication, 1920). Retrieved April 1, 2020, from http://www.gutenberg.org/files/33076/33076-h/33076-h.htm. The photograph from J. J. Gibson’s book: *The Perception of the Visual World* (Original date of publication, 1950, Figure 74, p. 184) was retrieved from a copy of the Utrecht University library. **(C,D)** Photographs are owned by the authors and the people depicted in the images gave consent for publication.

According to these scholars, psychological science is faced with a gloomy predicament: findings and results based on highly controlled and systematically designed laboratory experiments may not be a great discovery but only a *“mere laboratory curiosity”* (Gibson, 1970, pp. 426–427). As Anderson et al. (1999, p. 3) put it: *“A common truism has been that*… *laboratory studies are good at telling whether or not some manipulation of an independent variable causes changes in the dependent variable, but many scholars assume that these results do not generalize to the “real-world.”* The general concern is that, due to the ‘artificiality’ and ‘simplicity’ of the laboratory, some (if not many) lab-based experiments do not adequately represent the ‘naturality’ and ‘complexity’ of psychological phenomena in everyday life (see Figure 1). This problem has become familiar to psychologists as the ‘real-world or the lab’-dilemma (Hammond and Stewart, 2001). At the heart of psychology’s ‘real-world or the lab’-dilemma lies a pernicious methodological choice: *“Should it [psychological science] pursue the goal of generality by demanding that research be generalizable to “real life” (aka the “real-world”), or should it pursue generalizability by holding onto its traditional laboratory research paradigm?”* (Hammond and Stewart, 2001, p. 7).

Although psychological science is comprised of many specialized research areas, the goal to understand human cognition and behavior in the ‘real-world’ has become a critically acclaimed goal for psychologists and cognitive scientists of all stripes. Indeed, examples of the ‘real-world or the lab’-dilemma can be found not only in various ‘applied’ fields of psychology, such as ergonomics (Hoc, 2001), clinical (neuro)psychology (Wilson, 1993; Parsons, 2015), educational psychology (Dunlosky et al., 2009), sport psychology (Davids, 1988), marketing and consumer psychology (Smith et al., 1998), and the psychology of driving (Rogers et al., 2005), but also in the so-called ‘basic’ fields of psychological science, such as the study of perception (Brunswik, 1956; Gibson, 1979/2014), attention (Simons and Levin, 1998; Peelen and Kastner, 2014), memory (Banaji and Crowder, 1989; Neisser, 1991; Cohen and Conway, 2007), social cognition (Schilbach et al., 2013; Schilbach, 2015; Shamay-Tsoory and Mendelsohn, 2019; Osborne-Crowley, 2020), judgment-and-decision making (Koehler, 1996), and child development (Lewkowicz, 2001; Schmuckler, 2001; Adolph, 2019).

### The ‘Real-World Approach’: A Call for Ecological Validity

In the past decades, researchers have often discussed how they may overcome some of the limitations of laboratory-based experiments. Perhaps the largest common denominator of what we call the ‘real-world approach’ is a strong emphasis on ‘ecological validity.’ Over the past decades, the term ecological validity has made its appearance whenever researchers became concerned with the potential limitations of laboratory experiments (see e.g., Jenkins, 1974; Neisser, 1976; Banaji and Crowder, 1989; Aanstoos, 1991; Koehler, 1996; Smilek et al., 2006; Risko et al., 2012; Schilbach, 2015; Caruana et al., 2017; Shamay-Tsoory and Mendelsohn, 2019; Osborne-Crowley, 2020). As Neisser (1976, p. 33) famously put it:

“The concept of ecological validity has become familiar to psychologists. It reminds them that the artificial situation created for an experiment may differ from the everyday world in crucial ways. When this is so, the results may be irrelevant to the phenomena that one would really like to explain.”

The main problem, according to Neisser and many others, is that experiments in psychological science are generally *“lacking in ecological validity”* (Neisser, 1976, p. 7; Smilek et al., 2006; Shamay-Tsoory and Mendelsohn, 2019; Sonkusare et al., 2019). Aanstoos (1991, p. 77) even referred to this problem as the *“ecological validity crisis.”* To counter this problem, many researchers have called for studies with ‘more’ or ‘greater’ ecological validity. For example, Koehler (1996, p. 1) advocated for a *“more ecologically valid research program,”* Schilbach (2015, p. 130) argued for *“the inclusion of more ecologically valid conditions,”* and Smilek et al. (2006, p. 104) suggested that *“in order for results to generalize to real-world scenarios we need to use tasks with greater ecological validity.”* Clearly, ecological validity is regarded as an important feature of experimental research by researchers who pursue the ‘real-world approach.’ However, in our opinion, and we are not alone in this regard (see also Hammond, 1998; Araujo et al., 2007; Dunlosky et al., 2009), this notion of ecological validity has caused considerable confusion. To foreshadow some of our criticism of ecological validity, we will show that this concept has largely been detached from its original parentage (cf. Brunswik, 1949), and is now host to different interpretations guided by questionable assumptions (for a history, see Hammond, 1998). Worst of all, the concept is often wielded as a blunt weapon to criticize and dismiss experiments, even though researchers seldom make explicit what definition of ecological validity they use or by which set of criteria they have evaluated a study’s ecological validity (as previously pointed out by Hammond, 1998; Schmuckler, 2001; Dunlosky et al., 2009).

### The Big Umbrella of Ecological Validity

In past decades, the concept of ecological validity has been related to various facets of psychological research, for example, the ecological validity of stimuli (Neisser, 1976; Risko et al., 2012; Jack and Schyns, 2017), the ecological validity of tasks (Smilek et al., 2006; Krakauer et al., 2017), the ecological validity of conditions (Schilbach, 2015; Blanco-Elorrieta and Pylkkänen, 2018), the ecological validity of research settings (Bronfenbrenner, 1977; Schmuckler, 2001), the ecological validity of results (Eaton and Clore, 1975; Greenwald, 1976; Silverstein and Stang, 1976), the ecological validity of theories (Neisser, 1976), the ecological validity of research designs (Rogers et al., 2005), the ecological validity of methods (Banaji and Crowder, 1989), the ecological validity of phenomena (Johnston et al., 2014), the ecological validity of data (Aspland and Gardner, 2003), and the ecological validity of paradigms (Macdonald and Tatler, 2013; Schilbach et al., 2013). However, despite the popular usage of this term, specific definitions and requirements of ecological validity are not always clear.

A closer look at the literature suggests that different definitions and interpretations are used by researchers. Let’s consider some examples of the literature where researchers have been more explicit in their definitions of ecological validity. For example, Ashcraft and Radvansky (2009, p. 511) defined ecological validity as: *“The hotly debated principle that research must resemble the situations and task demands that are characteristic of the real-world rather than rely on artificial laboratory settings and tasks so that results will generalize to the real-world, that is, will have ecological validity.”* Another influential definition of ecological validity was given by Bronfenbrenner (1977), who defined ecological validity as *“the extent to which the environment experienced by the subjects in a scientific investigation has the properties it is supposed or assumed to have by the investigator”* (p. 516). In Bronfenbrenner’s view, a study’s ecological validity should not be predicated on the extent to which the research context resembles or is carried out in a ‘real-life’ environment. Instead, theoretical considerations should guide one’s methodological decisions on what type of research context is most appropriate given one’s focus of inquiry. For example, if one is interested in the behavioral responses of children when they are placed in a ‘strange situation’ then a laboratory room may be adequately suited for that particular research goal. However, if one is interested in how children behave within their home environment, then a laboratory room may not be the most suitable research context. As Bronfenbrenner (1977, p. 516) remarked: *“Specifically, so far as young children are concerned, the results indicate that the strangeness of the laboratory situation tends to increase anxiety and other negative feeling states and to decrease manifestations of social competence.”*

Ecological validity has also been used interchangeably with (or regarded as a necessary component of) ‘external validity’ (Berkowitz and Donnerstein, 1982; Mook, 1983; Hoc, 2001). The concept of external validity typically refers to whether a given study result or conclusion, usually obtained under one set of conditions and with one group of participants, can also be generalized to other people, tasks, and situations (Campbell, 1957). For example, in the literature on neuropsychological assessment and rehabilitation, ecological validity has primarily been conceptualized as *“*…*the degree to which clinical tests of cognitive functioning predict functional impairment”* (Higginson et al., 2000, p. 185). In this field, there has been much discussion about whether the neuropsychological tests used by clinicians accurately predict cognitive and behavioral impairments in everyday life (Heinrichs, 1990; Wilson, 1993). One major concern is that the test materials are either too abstract or too general to adequately represent the kind of problems that people with cognitive and neurological impairments encounter in their daily routines, for example, while cooking or buying food at the supermarket. In response, various efforts have been made to increase the ecological validity of neuropsychological tests, for example, by developing performance measures with relevance for everyday tasks and activities (Shallice and Burgess, 1991; Alderman et al., 2003), by combining and correlating tests results with behavioral observations and self-reports (Wilson, 1993; Higginson et al., 2000), and by using *Virtual Reality* (VR) applications to create test situations in which a patient’s cognitive and functional impairments are likely to be expressed (Parsons, 2015; Parsons et al., 2017).

## The Historical Roots of Ecological Validity

As we have seen, definitions and interpretations of ecological validity may not only differ among researchers, but also across various subfields within psychology. As such, it is not always clear how the concept should be interpreted. Interestingly, the term ecological validity used to have a very precise meaning when it was first introduced to psychological science by Brunswik (1949, 1952, 1955, 1956). Brunswik coined the term ‘ecological validity’ to describe the correlation between a proximal sensory cue (e.g., retinal stimulation) and a distal object-variable (e.g., object in the environment). In Brunswik’s terminology, ecological validity refers to a measure (a correlation coefficient) that describes a probabilistic relationship between the distal and proximal layers of an organism-environment system. According to Brunswik (1955): *“A correlation between ecological variables, one which is capable of standing in this manner as a probability cue for the other, may thus be labeled “ecological validity””* (p. 199). Brunswik (1952) believed psychology to primarily be a science of organism-environment relations in which the *“organism has to cope with an environment full of uncertainties”* (p. 22). In Brunswik’s ‘lens model’ (Brunswik, 1952), the ecological validities of perceptual cues indicate the potential utility of these cues for the organism to achieve its behavioral goals. Note that Brunswik’s concept of ecological validity is very different from how the term is generally used nowadays, namely to discuss and evaluate whether some laboratory-based experiments resemble and generalize to the ‘real-world’ (cf. Neisser, 1976; Smilek et al., 2006; Ashcraft and Radvansky, 2009; Shamay-Tsoory and Mendelsohn, 2019).

The erosion and distortion of Brunswik’s definition of ecological validity has been documented by several scholars (e.g., Hammond, 1998; Araujo et al., 2007; Holleman et al., in press). As explained by Hammond (1998), the original definition of ecological validity, as Brunswik (1949, 1952) introduced it, has been conflated with Brunswik’s ‘representative design’ of experiments (Brunswik, 1955, 1956). Representative design was Brunswik’s methodological program for psychological science to achieve generalizability of results. To achieve this, researchers should not only conduct proper sampling on the side of the subjects, by sampling subjects who are representative of a specific ‘target population’ (e.g., children, patients), but researchers should also sample stimuli, tasks, and situations which are representative of a specific ‘target ecology.’ As such, an experiment may be treated as a sample of this ‘target ecology.’ By virtue of sampling theory, researchers may then determine whether results can be generalized to the intended conditions. In short, representative design requires researchers to first specify the conditions toward which they intend to generalize their findings, and then specify how those conditions are represented in the experimental arrangement (Brunswik, 1956). For more in-depth discussions on representative design, see Hammond and Stewart (2001); Dhami et al. (2004), and Hogarth (2005).

## A Systematic Approach to Ecological Validity?

The current lack of terminological precision surrounding ecological validity is, to say the least, problematic. There seems to be no agreed upon definition in the literature, nor any means of classification to determine or evaluate a study’s ecological validity. This seems to be at odds with the relative ease by which researchers routinely invoke this concept to discuss the limitations and shortcomings of laboratory experiments. All the while, researchers seldom make clear how they have determined a study’s ecological (in)validity. As Schmuckler (2001, p. 419) pointed out: *“One consequence of this problem is that concerns with ecological validity can be raised in most experimental situations.”* To overcome these problems, several scholars have emphasized the need for a more systematic approach to ecological validity (Lewkowicz, 2001; Schmuckler, 2001; Kingstone et al., 2008; Risko et al., 2012). For example, Lewkowicz (2001, p. 443) wrote that: *“What is missing is an independent, objective, and operational definition of the concept of ecological validity that makes it possible to quantify a stimulus or event as more or less ecologically valid.”* According to Schmuckler (2001), ecological validity can be evaluated on at least three dimensions: (1) *the nature of the stimuli*; (2) *the nature of task, behavior, or response*; (3) *the nature of the research context*. Researchers have primarily discussed these dimensions in terms of their artificiality–naturality (e.g., Hoc, 2001; Schmuckler, 2001; Risko et al., 2012; Shamay-Tsoory and Mendelsohn, 2019; Sonkusare et al., 2019), and their simplicity–complexity (e.g., Kingstone et al., 2008; Peelen and Kastner, 2014; Lappi, 2015). As such, a general framework can be construed where stimuli, tasks, behaviors, and research contexts can be evaluated on a continuum of artificiality–naturality and simplicity–complexity (see also Risko et al., 2012; Lappi, 2015; Shamay-Tsoory and Mendelsohn, 2019; Osborne-Crowley, 2020). At one extreme is the laboratory, characterized by its artificiality and simplicity. At the other extreme is the ‘real-world,’ characterized by its naturality and complexity. According to this multidimensional framework, researchers may determine a study’s overall ecological validity by combining (e.g., averaging or summing) the main components of ecological validity (i.e., stimuli, tasks/behaviors, research context) in terms of their relative artificiality–naturality and simplicity–complexity. However, while many researchers have conceptualized ecological validity alongside these dimensions, we think there are several problems to consider. Since the dimensions of this framework are supposedly important to determine the ecological validity of experimental research, this then raises the question of how researchers can judge the artificiality–naturality and simplicity–complexity of particular experiments. This question will be explored in the following sections.

### Artificiality – Naturality

The contrast between ‘artificiality’ and ‘naturality’ is a particularly prominent point of discussion in the ‘real-world or the lab’-dilemma and when researchers talk about the ecological validity of experimental research practices (Hoc, 2001; Kingstone et al., 2008; Shamay-Tsoory and Mendelsohn, 2019). According to Hoc (2001, pp. 282–283), ‘artificial’ situations are *“those that are specifically designed for research”* and ‘natural’ situations are *“the target situations to be understood by research”*. Importantly, Hoc (2001) notes that this distinction is made from the perspective of the researcher. However, this artificiality–naturality distinction should also be considered from the subject’s point of view. For example, according to Sonkusare et al. (2019): *“naturalistic paradigms can be heuristically defined as those that employ the rich, multimodal dynamic stimuli that represent our daily lived experience, such as film clips, TV advertisements, news items, and spoken narratives, or that embody relatively unconstrained interactions with other agents, gaming environments, or virtual realities”* (p. 700). Furthermore, researchers have long recognized that artificiality arises when the experimental methods employed by researchers interfere with the naturality of the psychological phenomena one aims to study. Consequently, there is always an inherent trade-off between the degree of artificiality imposed by the experimental conditions and the naturality of the phenomena under scientific investigation (Brunswik, 1956; Barker, 1968; Banaji and Crowder, 1989; Kingstone et al., 2008; Risko et al., 2012; Caruana et al., 2017). However, as Winograd (1988) has previously remarked, it remains difficult to *“draw a line where artificiality ends and ecological validity*… *for real events begins”* (p. 18).

**FIGURE 2:**
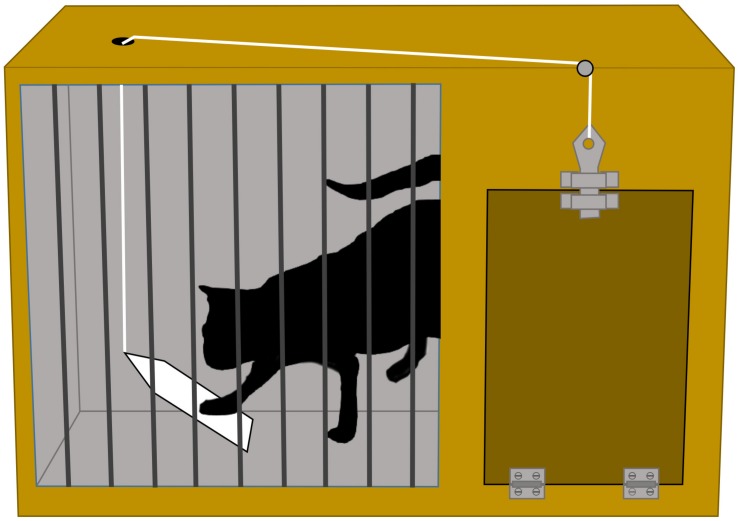
A ‘puzzle box’ devised by Thorndike (1899, 2017) to study learning behavior of cats. A hungry cat is placed in a box which can be opened if the cat pushes a latch. A food reward (‘positive reinforcer’) will be obtained by the cat if it figures out how to escape from the box. Thorndike discovered that after several trials, the time it takes the cat to escape from the box decreases. Experiments with puzzle boxes remain popular today to study the cognitive capacities of animals, for example, see Richter et al. (2016) for a study with octopuses. Copyright statement – Image created and owned by author IH and is based on E. L. Thorndike’s book: *Animal Intelligence* (Original date of publication, 1911, Figure 1, p. 30).

Interestingly, discussions on the naturality–artificiality of experimental methods have a long pedigree in psychological science. By the end of the 19th century, Thorndike (1899) and Mills (1899) already argued fiercely about what methodology should be favored to study the behavior of cats. Mills dismissed Thorndike’s work because of the artificiality of the experimental methods employed by Thorndike (see Figure 2), whereas Thorndike regarded the ethological approach favored by Mills as a collection of uncritical observations and anecdotes. Mills (1899, p. 264) wrote that: *“Dr. Thorndike*… *has given the impression that I have not made experiments, or ‘crucial experiments’*… *I may remark that a laboratory as ordinarily understood is not well suited for making psychological experiments on animals”*. Mills’ point was that: *“cats placed in small enclosures*…*cannot be expected to act naturally. Thus, nothing from about their normal behavior can be determined from their behavior in highly artificial, abnormal surroundings”* (Goodwin, 2015, p. 200). In response to Mills, Thorndike (1899, p. 414) replied: *“Professor Mills does not argue in concrete terms, does not criticize concrete unfitness in the situations I devised for the animals. He simply names them unnatural.”* Thorndike clearly did not accept Mills’ charge on the artificiality of his experimental arrangements to study the behavior of cats because Mills did not define what should be considered natural behavior in the first place.

We think that this historical discussion between Thorndike and Mills is illuminating, because it characterizes the heart of the discussion on ecological validity nowadays. Namely, what exactly did Mills consider to be ‘natural’ or ‘normal’ behavior? And how did Mills determine that Thorndike’s experiments failed to capture the ‘natural’ behavior of cats? Following Thorndike’s point on the matter, we think that researchers cannot readily determine the naturality–artificiality of any given experimental arrangement, at least not without specifying what is entailed by these ascriptions. As Dunlosky et al. (2009, p. 431) previously remarked: *“A naturalistic setting guarantees nothing, especially given that “naturalistic” is never unpacked – what does it mean?”.* Indeed, our survey of the literature also shows that the historical discussion between Thorndike and Mills is by no means a discussion of the past. In fact, we regularly encounter discussions on the ‘artificiality’ and ‘naturality’ of experimental setups, the presentation of stimuli, the behavior of participants, or the specific tasks and procedures used in experiments – not only in the literature, but also among our colleagues and reviewers. We must often ask for the specifics, because such remarks typically remain undefined by those who toss them around.

### Simplicity – Complexity

The contemporary framework of ecological validity also posits that the laboratory and the ‘real-world’ are inversely proportional in terms of their simplicity–complexity. Many researchers have lamented that laboratory experiments have a ‘reductionistic’ tendency to simplify the complexity of the psychological phenomena under study (e.g., Neisser, 1976; Kingstone et al., 2008; Shamay-Tsoory and Mendelsohn, 2019; Sonkusare et al., 2019). For example, Sonkusare et al. (2019, p. 699) stated that *“the ecological validity of these abstract, laboratory-style experiments is debatable, as in many ways they do not resemble the complexity and dynamics of stimuli and behaviors in real-life.”* But what exactly is meant by complexity? Let’s consider some examples from the literature. In the field of social attention, researchers have often used schematic images, photographs and videos of people and social scenes as stimuli to study the cognitive, behavioral, and physiological processes of face perception, gaze following and joint attention (Langton et al., 2000; Frischen et al., 2007; Puce and Bertenthal, 2015). However, in recent years, there has been considerable debate that such stimuli are not ‘ecologically valid’ because they do not *“capture the complexity of real social situations”* (Birmingham et al., 2012, p. 30). While we agree that looking at a photographic image of a person’s face is different from looking at a living and breathing person, in what ways do these situations differ in complexity? Do these scholars mean that looking at a ‘live’ person is more complex than looking at a picture of that person? Or do they mean that the former is more complex than the latter from the perspective of the researcher who wants to understand the cognitive, behavioral, and physiological processes of face perception and social attention?

**FIGURE 3:**
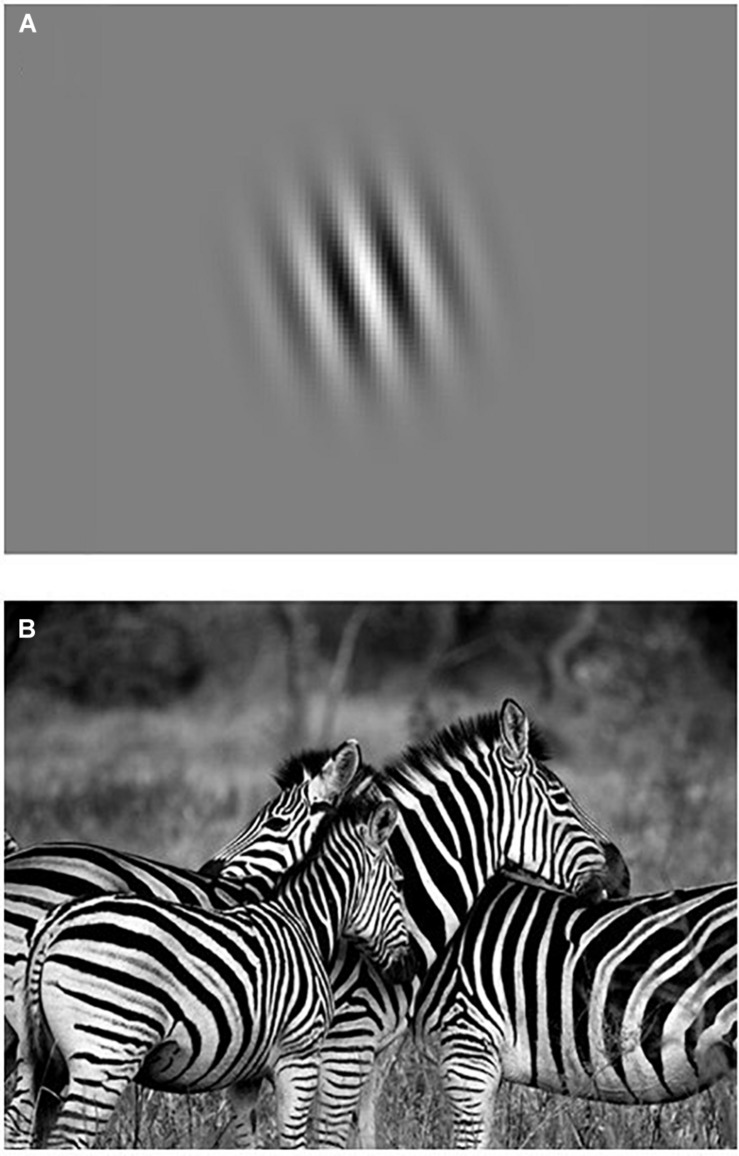
Are Gabor patches simple or complex compared to a picture of zebras? **(A)** A Gabor patch. **(B)** A photograph of zebras. The uniquely striped patterns of the zebra makes them most familiar to humans, whereas the question why zebras have such beautiful stripes remains the topic of much discussion among biologists, see e.g., Caro and Stankowich (2015) and Larison et al. (2015). Copyright statement – Images are used under the provision of the “fair use” U.S. Copyright Act 107 and Dutch Copyright Law Article 15a for non-profit purposes of research, education and scholarly comment. Image of Gabor patch was adapted from Todorović (2016, May 30). Retrieved April 1, 2020, from http://neuroanatody.com/2016/05/whats-in-a-gabor-patch/). Photograph of zebras was made by Ajay Lalu and has been made publicly available by the owner for non-profit purposes via *Pixabay*. Retrieved on April 1, 2020, from https://pixabay.com/nl/users/ajaylalu-1897335/.

To take another example, Gabor patches are often used as stimuli by experimental psychologists to study ‘low-level visual processing’ (see Figure 3). Experimental psychologists use Gabor patches as visual stimuli because they offer a high degree of experimental control over various stimulus parameters (e.g., spatial frequency bandwidths, orientation, contrast, size, location). Gabor patches can described with mathematical precision (i.e., *”Gaussian-windowed sinusoidal gratings,”* Fredericksen et al., 1997, p. 1), and their spatial properties are considered to be a good representation of the receptive field profiles in the primary visual cortex. While Gabor patches may be considered ‘simple’ to researchers who study the relation between low-level visual processing and neural activity in terms of orientation-tuning and hemodynamic response functions, they also point to the yet to be explained ‘complexity’ of the many possible relations between other cognitive processes and patterns of neural activity in the brain. On the other hand, a naïve participant (who likely has no clue about what researchers have discovered about low-level visual processing) may describe these Gabor patches as blurry, kind of stripy, zebra-like circles, and think that they are incredibly boring to look at for many trials while lying quietly in a MRI scanner.

Our point here is that simplicity–complexity is in the eye of the beholder. Who is to say what is more simple or complex? Physicists, computer scientists, information theorists, and evolutionary biologists have developed various definitions and measures of complexity (e.g., physical complexity, computational complexity, effective complexity, algorithmic complexity, statistical complexity, structural complexity, functional complexity, etc.), typically expressed in strictly mathematical terms (Edmonds, 1995; Gell-Mann, 1995; Adami, 2002). But what definitions and measures of complexity are used by psychologists and cognitive scientists? Researchers in psychological science seem to have more loosely used the term complexity, for example, to describe a wide range of biological, behavioral, cognitive, social, and cultural phenomena, which typically contain lots of *many’s* (i.e., many parts, many variables, many degrees of freedom). Researchers may refer to various phenomena as ‘complex’ because they are simply not (yet) understood, as in *“the brain is too complex for us to understand”* (Edmonds, 1995, p. 4). Yet, such intuitive notions of complexity, whether they are caused by ignorance or whether they are used to describe something’s size, number, or variety (Edmonds, 1995), are not very helpful to evaluate the simplicity–complexity of stimuli, tasks, and situations, nor do such notions provide any formula by which these components can be summed to determine the total ecological validity of a given study. According to Gell-Mann (1995, p. 16):

“As measures of something like complexity for an entity in the real-world, all such quantities are to some extent context-dependent or even subjective. They depend on the coarse graining (level of detail) of the description of the entity, on the previous knowledge and understanding of the world that is assumed, on the language employed, on the coding method used for conversion from that language into a string of bits, and on the particular idealized computer chosen as a standard.”

### The ‘Real World’ or the ‘Laboratory’: Psychology’s False Dilemma?

We have discussed several problems with how researchers have used the term ‘ecological validity’. In short, the concept of ecological validity has transformed significantly over the past several decades since it was introduced by Brunswik (1949). It has lost most of its former theoretical and methodological cohesion (for a history, see Hammond, 1998), and the definitions and requirements of ecological validity used by researchers nowadays are seldom made explicit. As such, some experiments may be regarded as ‘ecologically valid’ by one researcher while they can be casually dismissed as ‘ecologically invalid’ by others. A closer look at the literature suggests that many researchers seem to assume that everyone understands what is meant by this term, while in fact the concept of ecological validity is seldom defined. As such, the concept of ecological validity is primarily used nowadays to make hand-waving statements about whether some (lab-based) experiments resemble ‘real life,’ or whether some results obtained in the laboratory may or may not generalize to the ‘real-world.’

In our opinion, the contemporary framework of ecological validity eventually falls short of providing researchers with a tractable research program. Researchers seem to primarily base their judgments of ecological validity upon their own particular theoretical assumptions and considerations about the so-called artificiality–naturality and simplicity–complexity of experimental situations, typically in the absence of a more formal set of criteria. As such, while we certainly sympathize with the ‘call for ecological validity’, insofar it has motivated researchers to be critical about the limitations of experimental methods, we also think that the uncritical use of the term ecological validity has caused a lot of confusion, and in some cases has even been counterproductive. Perhaps the most problematic consequence of using the term ecological validity as an easy substitute for the ‘real-world’ was previously pointed out by Hammond (1998). He commented that:

*“There is, of course, no such thing as a “real-world.” It has been assigned no properties, and no definition; it is used simply because of the absence of a theory of tasks or other environments, and thus does not responsibly offer a frame of reference for the generalization”*.

In Hammond’s view, the aim to understand cognitive and behavioral functioning in the ‘real-world’ is basically pointless if one does not first define this notion of the ‘real-world.’ As such, researchers have locked themselves *“onto the horns of a false dilemma”* (Hammond and Stewart, 2001, p. 7). Thus, in order to talk sensibly about whether some results can also be generalized to particular situations beyond the experimental conditions in which those results were obtained, researchers first need to specify the range and distributions of the variables and conditions to which their results are supposed to be applicable. Since the notion of the ‘real-world’ patently lacks specificity, this phrase inevitably hampers researchers to specify the range and boundary conditions of cognitive and behavioral functioning in any given research context, and thus precludes one from getting at the context-specific and context-generic principles of cognition and behavior (see also Kruglanski, 1975; Simons et al., 2017).

### The Nature of the Environment?

Instead of trying to understand cognitive and behavioral functioning in the ‘real-world’, we completely agree with Hammond (1998) that the charge of researchers is to always specify and describe the particular context of behavior in which one is interested. Ultimately, the real challenge for researchers is to develop a theory of how specific environmental contexts are related to various forms of cognitive and behavioral functioning. But what constitutes a psychologist’s theory of the environment? Researchers in psychological science are typically concerned with the nature of the organism, yet, the nature of the environment and its relation to cognitive and behavioral functioning has received considerably less attention from a theoretical point of view (Barker, 1966; Heft, 2013). Interestingly, there have been several scholars who have dedicated themselves to precisely this question, and whose theories of cognition and behavior included a clear perspective on the nature of the environment.

According to Tolman and Brunswik (1935), the nature of the environment, as it appears to the organism, is full of uncertainties. The organism perceives the environment as an array of proximal ‘cues’ and ‘signs’ (i.e., information sources), which are the ‘local representatives’ of various distal objects and events in the organism’s environment. To function more or less efficiently, the organism needs to accumulate, combine, and substitute the information it derives from the available ‘cues’ and ‘signs,’ so that it can adequately adjust its means to achieve its behavioral goals (e.g., finding food or shelter). However, since the environment is inherently probabilistic and only partly predictable, the organism continually needs to adjust its assumptions about the state of the environment based on the available information sources. Another example is given by Barker (1968), whose concept of ‘behavior settings’ (see also Heft, 2001) is key in describing how the environment shapes the frequency and occurrence of human cognition and behavior. Important to behavior settings is that they are the product of the collective actions of a group of individuals. Their geographical location can be specified (e.g., the supermarket, the cinema, etc.), and they have clear temporal and physical boundaries (e.g., opening hours, a door to enter and exit the building). Behavior settings are ‘independent’ of an individual’s subjective experience, yet what goes on inside any behavior setting is characterized by a high degree of interdependency and equivalence of actions between individuals (e.g., most people who are inside a supermarket are shopping for groceries and people in cinemas are watching movies). Another ‘classic’ example of a theory of the environment can be found in J. J. Gibson’s book *The Ecological Approach to Visual Perception* (1979/2014). According to Gibson, there exists a strong mutuality and reciprocity between the organism and its environment. He introduced the concept of ‘affordances’ to explain how the inherent ‘meaning’ of things (i.e., functional significance to the individual) can be directly perceived by an individual perceiver and how this ‘information’ shapes the possibilities for potential actions and experiences. For example, a sufficiently firm and smooth surface may be walk-on-able, run-on-able, or dance-on-able, whereas a rough surface cluttered with obstacles does not afford such actions (Heft, 2001). In short, affordances are properties of an organism-environment system. They are perceiver-relative functional qualities of an object, event or place in the environment and they are dependent on the particular features of the environment and their relationships with the functional capabilities of a particular individual (for more in-depth discussions, see e.g., Heft, 2001; Stoffregen, 2003).

In order to describe and specify the environment and its relation to cognitive and behavioral functioning, we may draw on these scholars to guide us in a more specific direction. While we do not specifically recommend any of these perspectives, we think they are illuminating because these scholars motivate us to ask questions such as: What is the specific functional context of the cognitive and behavioral processes one is interested in? What are the relevant variables and conditions in this context given one’s focus of inquiry and level of analysis? What do we know or assume to know about the range and distribution of these variables and conditions? And how can these variables and conditions be represented in experimental designs to study specific patterns of cognitive and behavioral functioning? In order to answer some these questions, several researchers have emphasized the importance of first observing how people behave in everyday situations prior to experimentation. For example, Kingstone et al. (2008) advocated for an approach called *Cognitive Ethology*, which proposes that researchers should first observe how people behave in everyday situations before moving into the laboratory. In a similar vein, Adolph (2019) proposes that researchers should start with a rich description of the behaviors they are interested in order to first identify the “essential invariants” of these behaviors (p. 187).

## The Field of Social Attention: Away From the Real-World and Toward Specificity About Context

To exemplify how some of the ideas outlined above may be useful to researchers, we will apply these ideas to a research topic of our interest: social attention. The field of social attention, as briefly discussed previously, is primarily focused on how attention is influenced by socially relevant objects, events, and situations, most notably, interactions with other social agents. In recent decades, it has been argued extensively that the experimental arrangements used by researchers in this field need more ‘ecological validity’ in order to adequately study the relevant characteristics of social attention in the ‘real-world’ (Risko et al., 2012, 2016; Schilbach et al., 2013; Caruana et al., 2017; Macdonald and Tatler, 2018; Shamay-Tsoory and Mendelsohn, 2019). In the light of these concerns, several researchers have advocated to study *“real-world social attention”* (Risko et al., 2016, p. 1) and *“real-world social interaction”* (Macdonald and Tatler, 2018, p. 1; see also Shamay-Tsoory and Mendelsohn, 2019). One example of this is given by Macdonald and Tatler (2018). In this study, Macdonald and Tatler (2018) investigated how social roles given to participants influenced their social gaze behavior during a collaborative task: baking a cake together. Participants were either not given explicit social roles, or they were given a ‘Chef’ or ‘Gatherer’ role. Macdonald and Tatler (2018) showed that, regardless of whether social roles were assigned or not, participants did not gaze at their cake-baking partners very often while carrying out the task. After comparing their results with other so-called ‘real-world interaction studies’ (e.g., Laidlaw et al., 2011; Wu et al., 2013), the authors stated that: *“we are not able to generalize about the specific amount of partner gaze during any given real-world interaction”* (Macdonald and Tatler, 2018, p. 2171). We think that this statement clearly illustrates how the use of ‘real-world’ and ‘real life’ labels may lead to misleading and potentially counterproductive conclusions, as it seems to imply that ‘real-world interactions’ encompass a clearly defined category of behaviors. However, as argued previously, these so-called ‘real-world interactions’ are *not* a clearly defined category of behaviors. Instead, statements about generalizability need to be considered within a more constrained and carefully defined context (cf. Brunswik, 1956; Simons et al., 2017). This would make it more clear what researchers are talking about instead of subsuming studies under the big umbrella of the ‘real-world.’ For example, if the goal is to study how the cognitive and behavioral processes of social attention are influenced by different contexts and situations, researchers need to specify social gaze behavior as a function of these different contexts and situations.

Thus, instead of studying ‘real-world’ social attention in the context of ‘real-world’ social interactions, researchers should first try to describe and understand cake-baking attention (Macdonald and Tatler, 2018), sharing-a-meal attention (Wu et al., 2013), waiting-room attention (Laidlaw et al., 2011), walking-on-campus attention (Foulsham et al., 2011), Lego-block-building attention (Macdonald and Tatler, 2013), playing-word-games attention (Ho et al., 2015), interviewee-attention (Freeth et al., 2013), and garage-sale attention (Rubo and Gamer, 2018). By doing so, we may begin to understand the context-generic and context-specific aspects of attentional processes, allowing for a more sophisticated theory of social attention. These examples not only show the wide variety of behavioral tasks and contexts that are possible to study in relation to social attention, they also show that uncritical references to ‘ecological validity’ a.k.a. ‘real-worldliness’ are not very helpful to specify the relevant characteristics of particular behavioral contexts.

There are also good examples where researchers have been more explicit about the specific characteristics of social situations that they are interested in. Researchers in the field of social attention have, for example, tried to unravel the different functions of gaze behavior. One important function of gaze behavior is to acquire visual information from the world, however, within a social context, gaze may also signal important information to others which may be used to initiate and facilitate social interaction (see e.g., Gobel et al., 2015; Risko et al., 2016). In a series of experiments, researchers have systematically varied whether, and the degree to which social interaction between two people was possible, and measured how gaze was modulated as a function of the social context (Laidlaw et al., 2011; Gobel et al., 2015; Gregory and Antolin, 2019; Holleman et al., 2020). In other studies, researchers have been explicit about the task-demands and social contexts that elicit specific patterns of gaze behavior, for example, in the context of face-to-face interactions and conversational exchanges (Ho et al., 2015; Hessels et al., 2019). We think that, if researchers would try to be more explicit in their descriptions of task-demands and social contexts in relation to gaze, this may prove to be a solid basis for a more sophisticated theory of social attention, yet such work remains challenging (for a recent review, see Hessels, in press).

## Conclusion

We have argued that the ‘real-world approach’ and its call for ecological validity has several problems. The concept of ecological validity itself is seldom defined and interpretations differ among researchers. We believe that references to ecological validity and the ‘real-world’ can become superfluous if researchers would clearly specify and describe the particular contexts of behavior in which they are interested. This will be a more constructive way to uncover the context-specific and context-generic principles of cognition and behavior. As a final note, we hope that editors and reviewers will safeguard journals from publishing papers where terms such as ‘ecological validity’ and the ‘real-world’ are used without specification.

## Author Contributions

GH and RH drafted the manuscript. RH, IH, and CK edited and revised the manuscript.

## Conflict of Interest

The authors declare that the research was conducted in the absence of any commercial or financial relationships that could be construed as a potential conflict of interest.
